# Distinct transcriptomic response to Newcastle disease virus infection during heat stress in chicken tracheal epithelial tissue

**DOI:** 10.1038/s41598-021-86795-x

**Published:** 2021-04-02

**Authors:** Perot Saelao, Ying Wang, Ganrea Chanthavixay, Vivian Yu, Rodrigo A. Gallardo, Jack C. M. Dekkers, Susan J. Lamont, Terra Kelly, Huaijun Zhou

**Affiliations:** 1 Integrative Genetics and Genomics Graduate Group, University of California, Davis, CA 95616 USA; 2 Feed the Future Innovation Lab for Genomics to Improve Poultry, University of California, Davis, CA 95616 USA; 3 Department of Animal Science, University of California, Davis, CA 95616 USA; 4 School of Veterinary Medicine, University of California, Davis, CA 95616 USA; 5 Department of Animal Science, Iowa State University, Ames, IA 50011 USA

**Keywords:** Agricultural genetics, Gene expression

## Abstract

Newcastle disease (ND) has a great impact on poultry health and welfare with its most virulent (velogenic) strain. In addition, issues exacerbated by the increase in global temperatures necessitates a greater understanding of the host immune response when facing a combination of biotic and abiotic stress factors in poultry production. Previous investigations have revealed that the host immune response is tissue-specific. The goal of this study was to identify genes and/or signaling pathways associated with immune response to NDV (Newcastle disease virus) in the trachea, an essential organ where NDV replicate after the infection, by profiling the tissue specific transcriptome response in two genetically distinct inbred chicken lines when exposed to both abiotic and biotic stressors. Fayoumis appear to be able to respond more effectively (lower viral titer, higher antibody levels, immune gene up-regulation) and earlier than Leghorns. Our results suggest NDV infection in Fayoumis appears to elicit proinflammatory processes, and pathways such as the inhibition of cell viability, cell proliferation of lymphocytes, and transactivation of RNA, more rapidly than in Leghorns. These differences in immune response converge at later timepoints which may indicate that Leghorns eventually regulate its immune response to infection. The profiling of the gene expression response in the trachea adds to our understanding of the chicken host response to NDV infection and heat stress on a whole genome level and provides potential candidate genes and signaling pathways for further investigation into the characterization of the time-specific and pathway specific responses in Fayoumis and Leghorns.

## Introduction

Heat stress is a significant factor impacting the poultry industry due to climate change, and can result in reduced feed consumption^1^, digestion inefficiency^2^, impaired metabolism^3^, and reduced immune response^1,4^. Bartlett and Smith reported that heat stress decreased the total level of circulating IgM and IgG antibodies during primary and secondary immune response in broiler chickens^1^. In the chicken HD11 cell line, heat stress and lipopolysaccharide treatment resulted in an up-regulation of some immune-related genes in addition to heat shock proteins and chaperones^5^. Increasing global temperatures with climate change necessitate a greater emphasis on understanding the role that abiotic factors have on host physiology and immune response in poultry.‬ ‬‬‬‬‬‬‬‬‬‬‬‬‬‬‬‬‬‬‬‬‬‬‬‬‬‬‬‬‬‬‬‬‬‬‬‬‬‬‬‬‬‬‬‬‬‬‬‬‬‬‬‬‬‬‬‬‬‬‬‬‬‬‬‬‬‬‬‬‬‬‬‬‬‬‬‬‬‬‬‬‬‬‬‬‬‬‬‬‬‬‬‬‬‬‬‬‬‬‬‬‬‬‬‬‬‬‬‬‬‬‬‬‬‬‬‬‬‬‬‬‬‬‬‬‬‬‬‬‬‬‬‬‬‬‬‬‬‬‬‬‬‬‬‬‬‬‬‬‬‬‬‬‬‬‬‬‬‬‬‬‬‬‬‬‬‬‬‬‬‬‬‬‬‬‬Most studies of transcriptome response to pathogens are conducted in thermoneutral conditions, but those studies failed to take into consideration potential role of temperature plays in the immune response. Thus, it is essential to understand the bird’s tissue response to NDV under heat stress.‬‬‬‬‬‬‬‬‬‬‬‬‬‬‬‬‬‬‬‬‬‬‬‬‬‬‬‬‬‬‬‬‬‬‬‬‬‬‬‬‬‬‬‬‬‬‬‬‬‬‬‬‬‬‬‬‬‬‬‬‬‬‬‬‬‬‬‬‬‬‬‬‬‬‬‬‬‬‬‬‬‬‬‬‬‬‬‬‬‬‬‬‬‬‬‬‬‬‬‬‬‬‬‬‬‬‬‬‬‬‬‬‬‬‬‬‬‬‬‬‬‬‬‬‬‬‬‬‬‬‬‬‬‬‬‬‬‬‬‬‬‬‬‬‬‬‬‬‬‬‬‬‬‬‬‬‬‬‬‬‬‬‬‬‬‬‬‬‬‬‬‬‬‬‬‬‬‬‬‬‬‬‬‬‬‬‬‬ ‬‬‬‬

Virulent NDV is one of the most phenotypically virulent strains of avian paramyxovirus 1^6^ that is a constant threat to poultry trade and production worldwide^7^. The causative agent, Newcastle disease virus (NDV), is a paramyxovirus classified as avian orthoavulavirus 1, previously known as avian paramyxovirus 1 (APMV-1) [1]. First observed in Java, Indonesia in 1926, NDV has since spread globally and outbreaks continue to occur and be maintained in domestic poultry^8^. There are five pathotypes which categorize the various strains of the virus: viscerotropic velogenic; neurotropic velogenic; mesogenic; lentogenic; and asymptomatic enteric^8^. Virulent strains could result in up to 100% mortality in non-vaccinated NDV-infected chickens, while vaccination can help induce cross-protective antibodies^9^. However, limited access to vaccine and a lack of proper infrastructure in low-income nations limits the protection that vaccines can offer to prevent ND outbreaks. These limitations highlight the growing need for a better understanding of the underlying molecular mechanisms of how hosts respond to NDV infection, especially in the respiratory system where NDV replicates, in order to develop novel therapeutic strategies and improve current prophylactics against NDV.

Local mucosal immunity in the respiratory tract plays an important role in defense against respiratory pathogens. The trachea is one of important tissues/organs due to its role in early viral replication and immunity against various pathogens such as NDV, avian influenza, and mycobacterium^10–13^. Furthermore, airway epithelial cells are one of the primary sites of NDV replication and the transport of NDV infected epithelial cells is a significant driver of viral antigen presentation to antigen presenting cells^10^.

The purpose of this study was to investigate the host response to NDV infection during heat stress in two genetically distinct highly inbred chicken lines, Fayoumis and Leghorns. Fayoumis have been shown to be more disease resistant and heat stress tolerant than Leghorn birds^11,14–16^. By profiling the antiviral response in chicken tracheal tissue following NDV infection and heat stress we can further elucidate the underlying molecular mechanism of resistance to NDV infection under heat stress in poultry.

## Results

### Viral genome alignment in trachea transcriptome data

**Figure 1:**
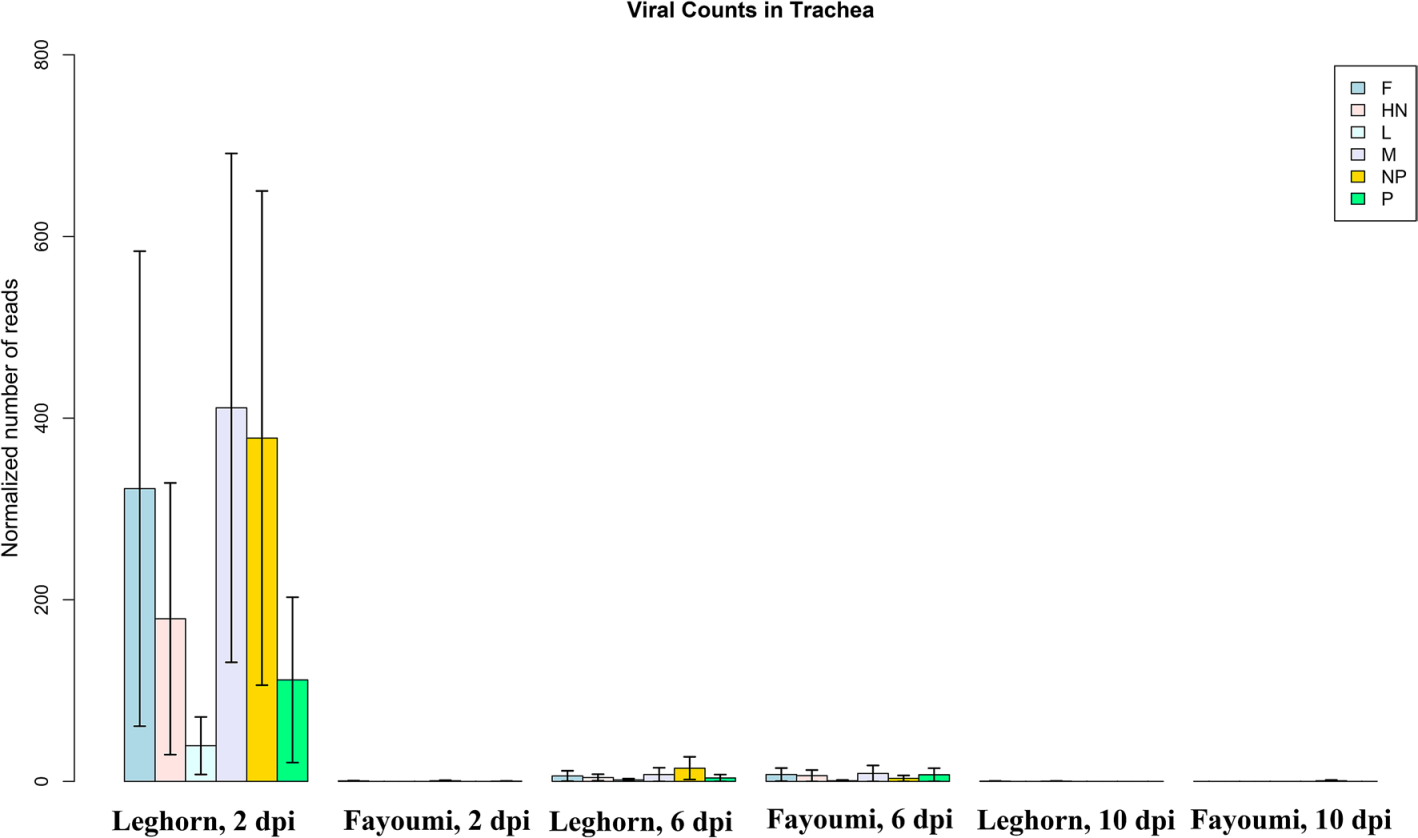
Normalized number of reads that aligned to specific gene segments of the La Sota Newcastle Disease viral genome found in the trachea; epithelium transcriptome: Fusion glycoprotein (F), Hemagglutinin-neuraminidase (HN), RNA-directed RNA polymerase L (L), Matrix protein (M), Nucleoprotein (NP), and Phophoprotein (P). Reads were extracted from treated individuals by genetic line and time point and aligned to the NDV La Sota genome. Bars indicate normalized number of reads with standard error. A higher number of reads aligned to the viral genome in Leghorn at 2 dpi, while at 6 dpi both lines observed detected viral transcripts, with no reads detectable in either line at 10 dpi.

Viral transcripts extracted from the tracheal transcriptome sequences of NDV-infected individuals from both lines were aligned to the NDV La Sota strain genome. In Fayoumis, NDV transcripts were only detected at 6 dpi, but not at 2 and 10 dpi, while Leghorns had significantly higher quantities of the NDV transcripts detected at 2 dpi, and with detectable quantities of the virus transcripts at 6 dpi and none at 10 dpi (Fig. 1).

### Differential gene expression from within-line comparisons

**Figure 2:**
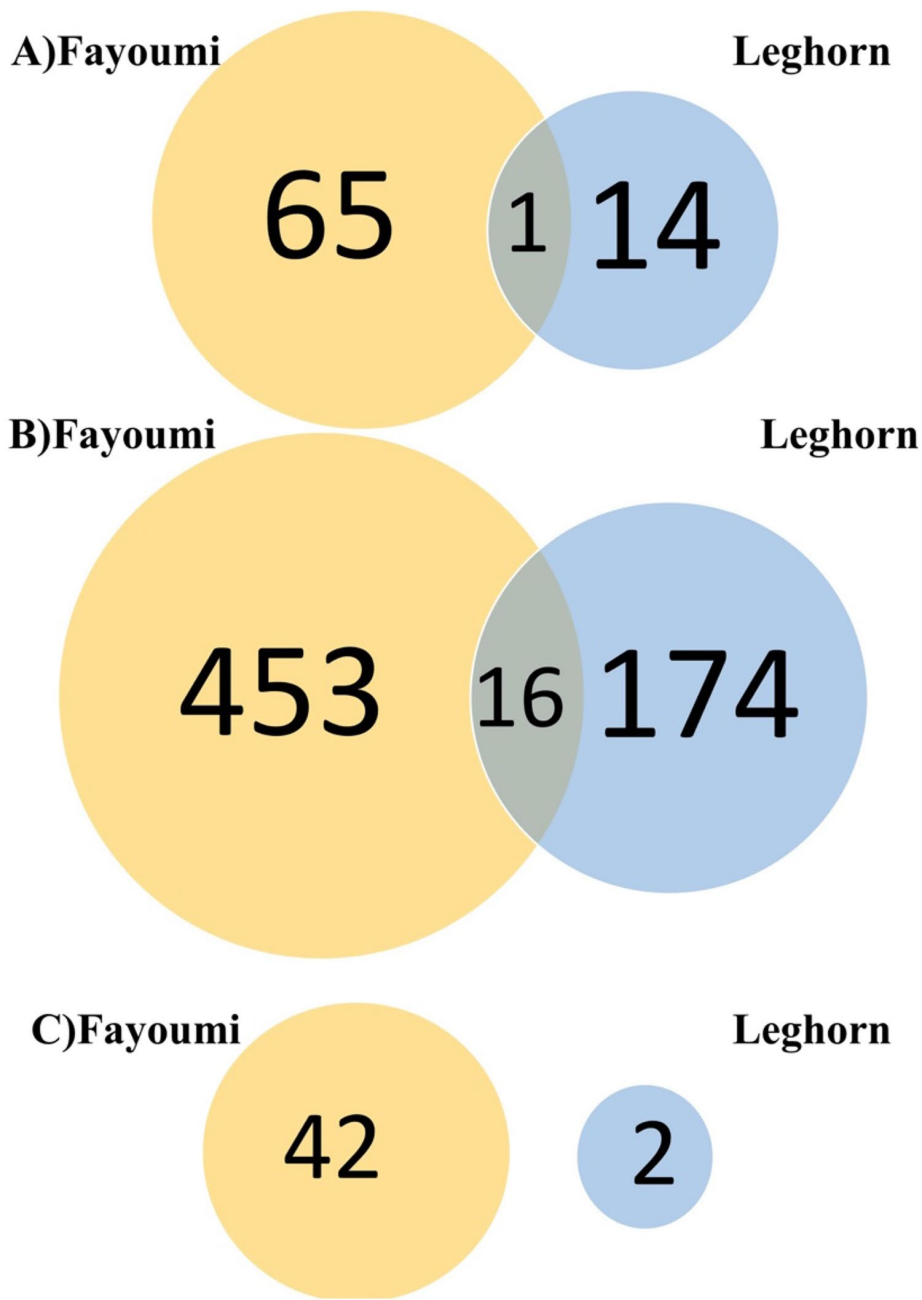
Venn diagrams displaying the number of overlapping differentially expressed genes (DEG) that overlapped between the two genetic lines by time point when comparing treated vs. non-treated birds. (**A**) Overlapped DEGs between Fayoumis and Leghorns at 2 dpi. (**B**) Overlapped DEGs between the two genetic lines at 6 dpi and (**C**) Overlapped DEGs between the two genetic lines at 10 dpi.

Within line comparisons between treated and non-treated individuals were performed in both Fayoumi and Leghorn chickens to identify differentially expressed genes (DEGs) in trachea at 2, 6, and 10 days post-infection (dpi) (Table 1). In Fayoumis at 2 dpi, there were 66 DEGs identified, with 45 genes significantly up-regulated and 21 genes down-regulated. Interestingly, at 6 dpi the number of up- and down-regulated DEGs increased in Fayoumis with 154 up-regulated and 315 down-regulated DEGs, then decreased at 10 dpi with 28 up-regulated and 14 down-regulated genes. For Leghorns at 2 dpi, only six DEGs were identified to be up-regulated and nine DEGs were down-regulated. At 6 dpi, there were 15 up-regulated and 175 down-regulated DEGs. However, at 10 dpi there were only two DEGs identified with one being up-regulated and the other down-regulated in Leghorns. Comparison within line of DEGs identified in response to NDV and heat demonstrated very little overlap between Fayoumis and Leghorns. One, 16, and zero DEGs were shared between the two lines at 2, 6, and 10 dpi, respectively (Fig. 2). The single gene at 2 dpi was down regulated for both lines. All but a single gene of the overlapped DEGs was up-regulated in Fayoumi. In Leghorns only two genes were down-regulated in the overlapped DEGs.

**Table 1:** Numbers of differentially expressed genes that were up-regulated and down-regulated between treated and non-treated groups by genetic line and time post-infection.

Line	Differentially expressed genes		Days post-infection
	Up-regulated	Down-regulated	
Fayoumi	45	21	2
Fayoumi	154	315	6
Fayoumi	28	14	10
Leghorn	6	9	2
Leghorn	15	175	6
Leghorn	1	1	10

### Pathway analysis of differentially expressed genes

Ingenuity Pathway Analysis (IPA) using the significantly differentially expressed genes between the treated and non-treated birds was used to identify gene networks and signaling pathways enriched at each timepoint. A summary of the top five canonical pathways identified by IPA analysis is presented in Table 2. Overall, Fayoumis had 50, 205, and six significantly enriched pathways at 2, 6, and 10 dpi, respectively. Among the top canonical pathways enriched in Fayoumis, the majority of these pathways were involved in acute phase and metabolitic processes (Table 2). Leghorn had only six significantly enriched pathways at 2 dpi, 65 pathways at 6 dpi, and none at 10 dpi (low number of DEGs). The enriched pathways at 2 dpi were related to cellular processes such as Ethanol degradation, retinoate biosynthesis, and serotonin degradation. However, at 6 dpi the most enriched pathways appear to highly involve the immune response. T Helper Cell Signaling and immune cell activation appear to demonstrate the activation of the host immune response in Leghorns.

**Table 2:** Top five most significant canonical pathways identified by ingenuity pathway analysis among differentially expressed genes from within line comparisons by timepoint.

Line, timepoint	Ingenuity canonical pathways	− log (p-value)	z-score
Fayoumi, 2 dpi	Role of tissue factor in cancer	5.14	
	Acute phase response signaling	4.5	0.447
	Circadian rhythm signaling	4.32	
	Extrinsic prothrombin activation pathway	3.3	
	Glucocorticoid receptor signaling	3.14	
Fayoumi, 6 dpi	Production of nitric oxide and reactive oxygen species in macrophages	6.68	
	CD40 signaling	5.12	
	Cholecystokinin/gastrin mediated signaling	5.03	1
	IL-8 signaling	4.91	0.905
	B cell activating factor signaling	4.9	− 0.447
Fayoumi, 10 dpi	Aldosterone signaling in epithelial cells	2.12	
	Protein ubiquitination pathway	1.75	
	tRNA charging	1.52	
	iNOS signaling	1.46	
Leghorn, 2 dpi	Circadian rhythm signaling	4.45	
	Retinoate biosynthesis I	2.03	
	Ethanol degradation II	1.99	
	Noradrenaline and Adrenaline degradation	1.96	
	Serotonin degradation	1.67	
Leghorn, 6 dpi	Th1 and Th2 activation pathway	12.5	
	Communication between innate and adaptive immune cells	11.9	
	Th2 pathway	11.1	1.414
	iCOS-iCOSL signaling in T helper cells	10.7	1.89
	CD28 signaling in T helper cells	10.4	2.646

Z-score > 0 indicates a pathway predicted to be activated, and Z-score < 0 predicts the pathway to be inhibited. No value listed indicates the analysis was unable to predict pathway activity.

**Figure 3:**
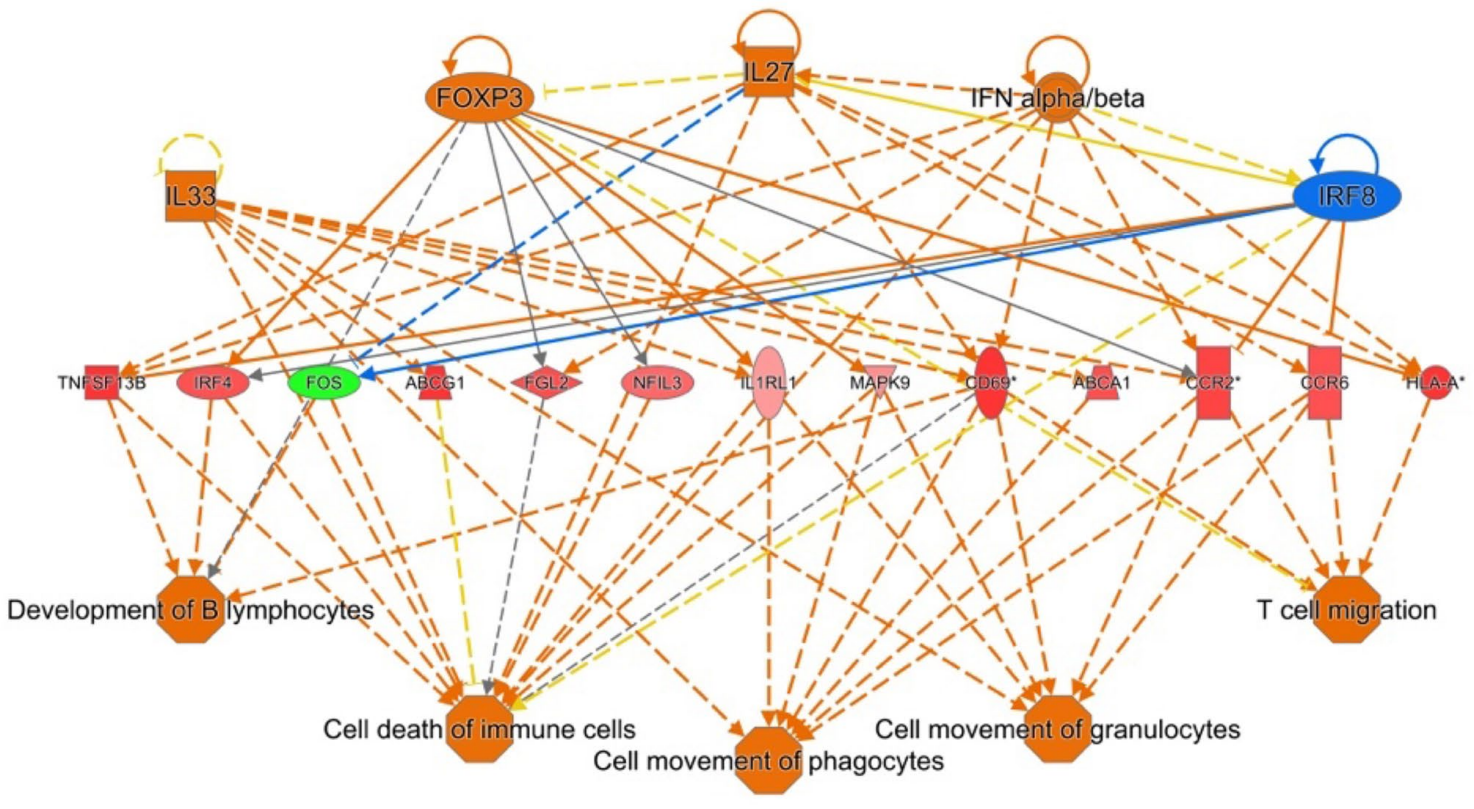
Top regulatory effect predicted by Ingenuity Pathway Analysis within Fayoumis at 6 dpi comparison. Blue (inhibited) and orange (activated) lines represent relationships with upstream regulators at the top of the figure with differentially expressed genes at center, and diseases/functions. Genes in green indicated down-regulation, while red signifies up-regulation.

To gain further insight into the potential physiological outcomes from these enriched pathways, IPA’s Regulator Effects predictor was used to predict upstream regulators and functional downstream outcomes. At 2 dpi in Fayoumi, we observed an overall inhibition of upstream regulators resulting in the inhibition of cell viability, cell proliferation of lymphocytes, and transactivation of RNA (Supplemental Fig. S1). However, at 6 dpi there appears to be a substantial shift towards activation of several upstream regulators leading to activation of process such as B lymphocyte development, and granulocyte and phagocyte migration (Fig. 3). No significant regulator effects were predicted within Fayoumis at 10 dpi and Leghorns at 2 dpi. For Leghorns, however, 6 dpi was the only timepoint with a significant regulator predicted which, similar to Fayoumi at 6 dpi, demonstrated significant activation of immune cells and related processes (Supplemental Fig. S2).

## Discussion

This is part of a large project under Feed the Future Innovation Lab for Genomics to Improve Poultry (http://gip.ucdavis.edu), whose overall goal of the program is to genetically enhance disease resistance to vNDV infection in African poultry. Two parallel studies (only biotic stressor, and a combination of biotic and abiotic stressors (current study) using the same genetic lines have been conducted. Transcriptome analysis of the within-line differences in the trachea revealed novel patterns of host response to NDV infection and heat stress across a time series in two unique genetic lines of chickens. We hypothesized that the unique genetic background and natural history of the two genetic lines would provide insights into the potential genetic mechanisms regulating immune response and disease resistance to NDV infection under heat stress in poultry. The non-pathogenic La Sota strain used in this study is commonly used as the vaccine strain when inoculating chickens against NDV and was utilized in this study due to its ability to still produce an immune response without a high mortality. The strain used in this study did not elicit any associated clinical symptoms. Phenotypically, the two lines have demonstrated unique physiological differences in response to heat stress^14^ and differences in viral titer and antibody levels with and without heat stress^11^. In these studies, the viral titer was confirmed through qRT-PCR at 2 dpi and 6 dpi, these results showed a significantly higher load of virus in Leghorn than in Fayoumis at both 2 and 6 dpi^17,18^. Additionally, ELISA results at 10 dpi showed differences in the antibodies produced by the two lines, and Fayoumis generated more anti-NDV antibodies than compared to Leghorns^17,18^. At 2 dpi, we observed a moderately high number of DEGs in Fayoumis (66) compared to Leghorns (15). In addition, the majority of the DEGs in Fayoumi at this timepoint were up-regulated (45) compared to down-regulated (21), while Leghorns found approximately equal numbers of up and down-regulated genes (6 and 9). The robust initial immune response seen in Fayoumis has been previously observed in the spleen of heat treated and lipopolysaccharide (LPS) treated Fayoumis potentially demonstrating a link to immune related processes^5^. Furthermore, three candidate genes Solute Carrier 29A, Cytochrome 8B1, and NF kappa B inhibitor alpha (*SLC29A, CYP8B1*, and *NFKBIA*) were significantly up-regulated within Fayoumis at 2 dpi and are regulators of the acute phase response of the immune system^19^. This pathway was predicted to be significantly activated at 2 dpi due to the up regulation *of SLC29A, CYP8B1*, and *NFKBIA* within the acute phase response signaling pathway**.** This more substantial acute immune response elicited early in the infection in Fayoumis could potentially prevent NDV replication in trachea and may explain the lack of viral transcripts detected in the Fayoumi trachea, while Leghorns had a significantly larger quantity of the NDV viral transcripts at 2 dpi. However,trace amounts of viral transcripts were identified in the trachea of Fayoumis at 6 dpi, with no difference between lines, which may have been due to recurrent infection or the transmission of the virus from within the infected birds. Viral loads were previously reported at 6 dpi in Fayoumis, so it may have been possible that trace quantities of the virus was able to make its way to trachea^17,18^.

The largest gene regulation changes in the host response to the treatment (NDV + heat stress) across different time points was observed at 6 dpi for both genetic lines. Both Fayoumis and Leghorns had a substantial number of DEGs (469 and 190, respectively). Despite the overall number of DEGs differing between the two genetic lines, both lines had significant enrichment of immune- related genes. Interestingly, *Interferon Regulatory Factor 4* (*IRF4*), which is involved in the interferon (IFN) activation of the innate defense response^20^ and has been shown to control and regulate the B-cell proliferation in chickens^21^, was significantly up-regulated in both genetic lines but at a much higher level in Fayoumis than Leghorns with a log_2_(fold change) (LFC) of 2.97 over 1.398. This substantial difference in the expression of critically important immune genes may potentially explain the disease resistance differences observed between the two genetic lines in response to NDV infection during heat stress.

Very few shared DEGs between the two lines were observed. At 2 dpi, only one gene, *Period Circadian Regulator 3* (*PER3*) was shared between the two genetic lines. Interestingly, this gene was down regulated in both Fayoumis and Leghorns (LFC = − 1.09 and − 1.27), which suggest its potential function as a general stress response gene in chickens. At 6 dpi the number of overlapping DEGs increased to 16, although this is a relatively small number of DEGs shared between the two lines considering the larger number of DEGs identified at 6 dpi. The increase in overlap of DEGs, however, appeared to be shared among several immune response genes such as *Interferon Factor 4IRF4* and several *Chemokine Receptor (CCR)* genes, which suggest these genes are important for the host to generate a general immune response to NDV infection. These CCR or chemokine receptor genes are important for the migration of various cell types into the inflammatory sites. These differentially expressed genes may explain the difference in response observed between Fayoumis and Leghorns based on the magnitude of response of these genes and highlight the importance of these genes to the immune response generally.

Pathway analysis of the Fayoumis at 2 dpi identified a robust enrichment in immune related pathways such as: *Interleukin signaling (IL-12)*, NF-κB activation by viruses, Toll-like Receptor, and Acute Phase Response signaling pathways. Interleukin signaling is the primary driver of the immune cell differentiation program that regulates the survival, proliferation, and maturation of leukocytes within the host^22^. The NF-κB pathway has long been considered the prototypical inflammatory signaling pathway, with complex roles within pro- and anti-inflammatory processes^23^. Activation of the NF-κB pathway can result in recruitment of leukocytes to the site of infection and release additional proinflammatory cytokines to rapidly signal the immune response through Toll-like receptors detection of microbes^23^. We speculate that the more immune-related pathways enriched at 2 dpi in Fayoumis compared to Leghorns likely reflects the Fayoumi birds’ enhanced ability for early response to NDV during heat stress. In contrast, there were few signaling pathways enriched in Leghorns at 2 dpi, and none of the pathways enriched in this study were related to immune response. Activation of strong innate response at early time of NDV infection plays a critical role in shaping the outcome of the infection. The above results suggest that Fayoumis were able to mount a more robust immune response earlier in infection and thus generated more enhanced local resistance to NDV than Leghorns. For example, Acute phase response signaling, significantly enriched at 2 dpi in Fayoumis, is involved with rapid inflammatory response to provide protection against infection using non-specific defense mechanisms^19^. Regulator effects predicted in IPA demonstrated a down-regulation and overall inhibition of cell proliferation and cell viability. Studies in mice have observed a similar pattern of inhibition in cell proliferation during early stages of infection, likely due to the virus attempting to suppress the adaptive immune response of the host. However, the predicted activation of IL and acute phase signaling suggest that Fayoumis are primarily eliciting an innate inflammatory response to NDV^19,23^. This is further demonstrated by the activation of other general pathogen inflammation pathways such as NF-κB activation and IL-12 signaling.

Although the response to NDV and heat stress at 2 dpi differed between the two genetic lines, the transcriptome at 6 dpi presented a more similar response from the two genetic lines with respect to pathways that were enriched at this timepoint related to the immune response. At 6 dpi, both Fayoumis and Leghorns had a large number of enriched signaling pathways (205 and 65, respectively). Fayoumis appeared to be stimulating interleukin and NF-κB driven response with significantly activated signaling pathways, such as the IL-8 and NF-κB signaling pathways. In addition, many of these pathways in Fayoumis were predicted to be activated (80) compared to down regulated (23). On the contrary, the Leghorn birds had only activated pathways predicted, and many of these pathways were involved with T cell and T lymphocyte-mediated signaling. The signaling pathway enrichment in immune response pathways in both genetic lines indicates that the 6 dpi timepoint appears to be a critical timepoint for the host to be eliciting a strong immune response by actively recruiting immune cells to respond to the presence of the virus within the trachea. Regulator effect prediction similarly detected an overall activation in upstream regulators and down-stream immune related processes in both Fayoumis and Leghorns. However, NDV viral titer differences in tears between Fayoumis and Leghorns observed in the previous studies may suggest that the earlier activation of immune related pathways in Fayoumis may contribute to relative resistance to NDV compared to Leghorns while under heat stress^17,18^. Similarly, viral transcripts obtained at 6 dpi between the two lines shows very low levels of the virus present in both lines suggesting that both lines may rely more heavily on an earlier response to clear the virus at 2 dpi rather than 6 dpi. Alternatively, Fayoumis may be able to convey improved resistance due to the utilization of a heavily interleukin (IL) driven response. Previous studies suggest that although there exists “cytokine redundancy”, the ‘availability’, ‘context’, and ‘nature of the signal’ can derive unique and different activation of specific cytokines^24^. While activation of the immune response was observed by both genetic lines, there may be a line specific response that was unique due to the activation of distinct biological responses in the two genetic lines.

Only six significantly enriched pathways were identified at the 10 dpi timepoint in Fayoumis, and no pathways were enriched in Leghorns. This was likely mostly due to the fact that very few DEGs were found between treatment conditions and may be suggesting that both lines were returning to homeostatic levels and resolving the infection.

In a parallel study performed by Deist et al. 2017 using the same experimental procedure with the same genetic lines but under thermoneutral conditions, the authors found similar results of lower viral titer and higher antibody response to NDV in Fayoumis than Leghorns in other tissues^17,18,25^. Similar to the results presented in this study, both genetic lines had a more robust response to NDV infection at earlier timepoints and very little response observed at 10 dpi. However, in the presence of heat stress it appears that Fayoumis mounted a stronger (in terms of both DEGs and enriched pathways) at 6 dpi whereas in the absence of heat stress the response was the most robust at 2 dpi. In both studies, however, Leghorns appeared to have significantly higher amounts of detected viral transcripts in the trachea at 2 dpi, and reduced activation of immune related pathways. The results highlighted in this study provide insight into the effects of heat stress on NDV infection and the importance of understanding abiotic factors that can influence the immune system.

Previous investigations into the lung and Harderian gland highlight unique and tissue specific processes unique to each tissue type while under heat stress^17,18^ or thermal neutral conditions^26^. Fayoumis appear to activate gene expression as early as 2 dpi to infection compared to Leghorns based on the number of DEGs and signaling pathways identified. We would expect this alternation in gene expression would correlate with the improved heat and disease tolerance demonstrated in Fayoumis based on their ability to maintain lower viral titers. However, activation of proinflammatory pathways were observed at both lines at 2 dpi as expected. At 6 dpi, the response observed across all three tissues diverged significantly in terms of the numbers of DEGs identified. Fayoumis had very few DEGs found in the Harderian gland and lung, while the trachea had substantially more DEGs than either tissue. One potential explanation for the higher number of DEGs found in the trachea was that the virus had established within that tissue and was still replicating at detectable levels at this time point. This was not the case for the Harderian gland and lung, where no virus was detected at 6 dpi in Harderian gland and no virus was detected at all time points within the lung. Interestingly, Leghorns appeared to have larger numbers of DEGs in the Harderian gland and trachea, but fewer DEGs in the lung. The low number of DEGs found in the Lung was contradictory to the proteomic data which observed a sharp increase in proteins altered due to NDV infection at 6 dpi. The differences observed in the lung compared to the other tissues may be due to the fact that the virus was less prevalent and no longer replicating in the lung, or that the virus was cleared before reaching the lung, and that the expression profile of the host has adapted accordingly.

## Conclusion

In this study, the tracheal epithelial layer of cells was used for the global transcriptome analysis of the chicken host response to NDV during heat stress as it is one of the primary tissues for infection by poultry respiratory viruses. The analysis performed in this study has provided novel insights into the changes and response of two unique lines chickens and the various signaling pathways that may explain the differences in response patterns between the two lines. Infection by NDV appeared to elicit proinflammatory processes much earlier in Fayoumis than in Leghorns. However, both genetic lines eventually mount a robust response at 6 dpi which may indicate that Leghorns eventually activate its immune response to infection. The effective timing and response to NDV over the course of infection needs to be further investigated in order to properly understand what specific cellular and molecular mechanisms contributed to the difference in resistance to NDV between the two lines. The information gained through this study have provided informative and insightful clues for further development of novel therapeutics and prevention strategies to NDV during heat stress in poultry.

## Materials and methods

### Experimental populations and design

The experimental design of this study has been previously described by Wang et al. 2018^14^. In brief, Fayoumi (M15.2) and Leghorn (GHs 6) chicken lines from Iowa State University poultry research and teaching facility (Ames, IA) were used. On day of hatch, 56 Fayoumi and 55 Leghorn chicks were transported from Iowa State University to Davis, CA. Upon arrival, the chicks were housed in temperature and humidity-controlled chambers at the biosafety level 2 animal facility at the University of California, Davis. Twenty-five individuals from each genetic line were randomly selected and housed in a separate chamber to be used as the control group, and the rest as the treatment group. From day 1 to day 13 both groups were reared at 29.4 °C and 60% humidity. At 14 days of age, the treatment group was exposed to 38 °C for 4 h, then decreased to 35 °C and maintained at this temperature until the conclusion of the trial. The control group was maintained at 25 °C. On day 21 the heat-treated birds were inoculated with 200 μL 10^7^ EID_50_ of the La Sota strain of NDV through both ocular and nasal passages. The control group was mock inoculated with 200 μL of 1 × phosphate-buffered saline (PBS). At 2, 6, and 10 days post-infection (dpi), 4 birds per treatment group per genetic line were randomly selected and euthanized with CO_2_ and tracheal epithelial tissue was harvested by scraping the trachea then quickly placed into RNA*later* (ThermoFisher Cat#AM7024) and kept at − 80 °C. The experiment's procedures were performed according to the guidelines approved by the Institutional Animal Care and Use Committee at the University of California, Davis (IACUC #17853).

### RNA-isolation and library construction

The methods for RNA and library construction have been previously described in Wang et al. 2018^14^ and Saelao et al. 2018^18^. In brief, total RNA was extracted from the scraping of the tracheal epithelial layer of four individuals per treatment (non-infected and infected) and genetic line (Fayoumi and Leghorn) for each of the three time points. The trachea was homogenized in ice cold TRIzol (ThermoFisher Cat#15596026) and processed using a standard phenol:chloroform method and precipitated in 100% ethanol. The RNA pellet was then dissolved into water and treated with DNase I (ThermoFisher Cat#EN0521). Strand specific RNA library preparation was prepared exactly as stated in the NEBNext Ultra Directional RNA Library Prep Kit for Illumina (NEB Cat#E7420S). Library validation and quantification was done using the Agilent Bioanalyzer High Sensitivity Kit (Agilent Cat#5067-4626) and Qubit dsDNA HS Assay kit (ThermoFisher Cat#Q32854). The 100 base pair, paired-end sequencing was performed on the Illumina HiSeq2500 system with a minimum sequencing depth of 30 million reads.

### Data analysis

Statistical data analysis and visualization were performed using the software R with standard packages^27^. Four major factors were included for analysis: condition (treated, non-treated), line (Leghorn, Fayoumi), sex (male, female), and time point (2, 6, and 10 dpi). Data at each time point consisted of 16 individuals (4 per treatment and per genetic line). Raw reads from RNA-seq were trimmed using FastQC^28^ to remove duplicates, reads with base quality scores < 30, and adapter contamination. These reads were then aligned using STAR^29^ to the galGal5 reference genome and Ensembl annotation using default settings. Gene counts for chicken and viral transcripts aligned to the LaSota NDV genome were calculated using HTSeq^30^ and differential gene analysis was done using edgeR^31^. The statistical model design included the effects of line, condition, sex, and time point. Genes were identified as differentially expressed (DEGs) if they had a false discovery rate (FDR) < 0.05, and an average transcript count > 10. Pathway analysis and regulator effect prediction using the DEGs of within line contrasts was performed using Qiagen’s Ingenuity Pathway Analysis software^32^. Sequence data is available through NCBI’s Sequence Read Achieve through project number: PRJNA662738.

### ARRIVE guidelines

This study was carried out in compliance with the ARRIVE guidelines.

### Ethics approval

The experiment's procedures and animal handling were approved by the Institutional Animal Care and Use Committee at the University of California, Davis (IACUC #17853). All experiments were performed according to guidelines and policy set by that this committee.

## Supplementary Information

Supplementary Information

## Data Availability

Sequence data is available through NCBI’s Sequence Read Achieve through project number: PRJNA662738.
