# Supplementary material for: Distinct transcriptomic response to Newcastle disease virus infection during heat stress in chicken tracheal epithelial tissue
Source: Sci Rep. 2021 Apr 2;11:7450. doi: 10.1038/s41598-021-86795-x (PMC8018950; doi:10.1038/s41598-021-86795-x)
Supplement: Supplementary file 1 — Supplementary Information [file 41598_2021_86795_MOESM1_ESM.pptx]

## Slide 1
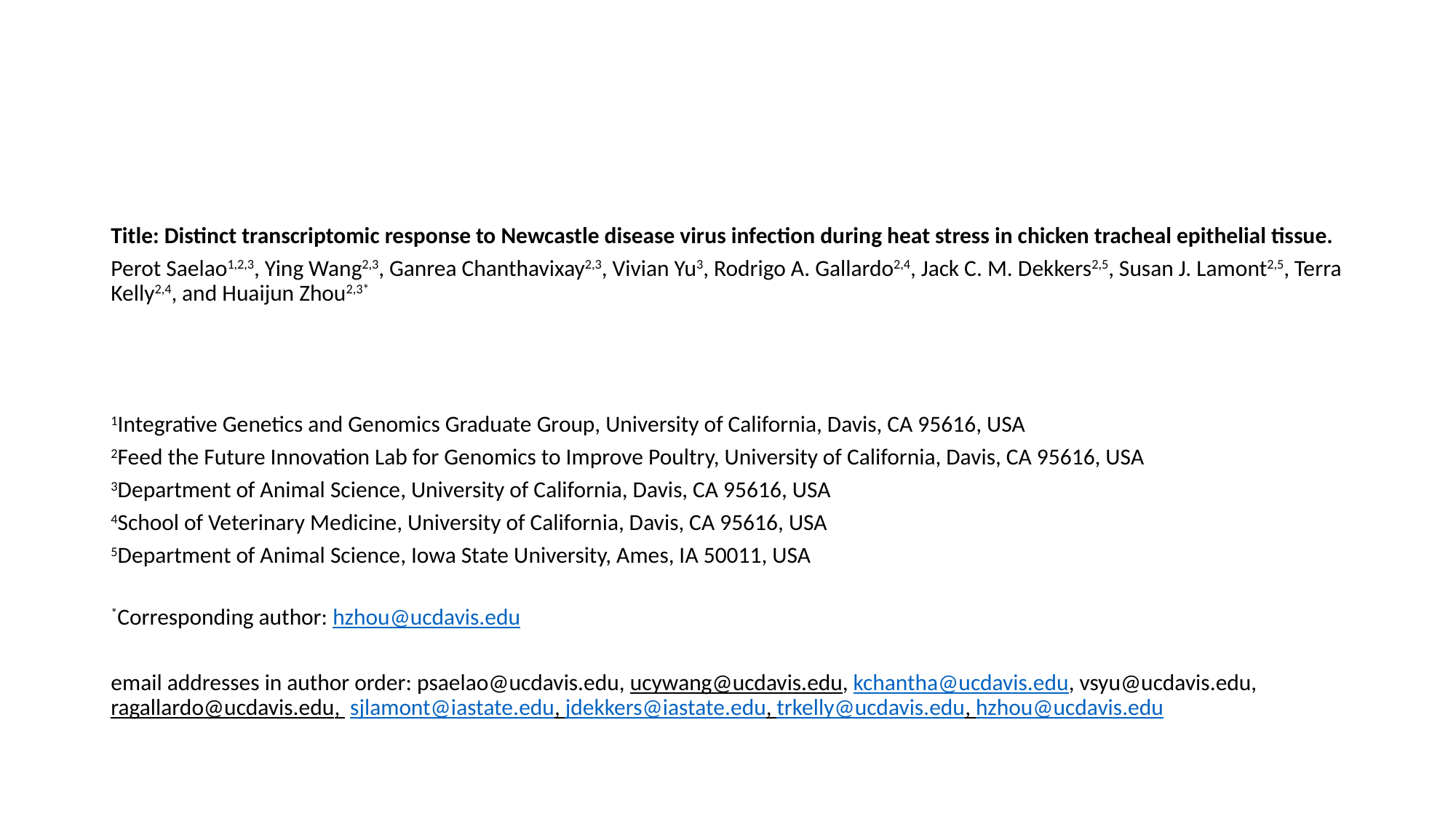

Title: Distinct transcriptomic response to Newcastle disease virus infection during heat stress in chicken tracheal epithelial tissue.
Perot Saelao1,2,3, Ying Wang2,3, Ganrea Chanthavixay2,3, Vivian Yu3, Rodrigo A. Gallardo2,4, Jack C. M. Dekkers2,5, Susan J. Lamont2,5, Terra Kelly2,4, and Huaijun Zhou2,3*
1Integrative Genetics and Genomics Graduate Group, University of California, Davis, CA 95616, USA
2Feed the Future Innovation Lab for Genomics to Improve Poultry, University of California, Davis, CA 95616, USA
3Department of Animal Science, University of California, Davis, CA 95616, USA
4School of Veterinary Medicine, University of California, Davis, CA 95616, USA
5Department of Animal Science, Iowa State University, Ames, IA 50011, USA
*Corresponding author: hzhou@ucdavis.edu
email addresses in author order: psaelao@ucdavis.edu, ucywang@ucdavis.edu, kchantha@ucdavis.edu, vsyu@ucdavis.edu, ragallardo@ucdavis.edu,  sjlamont@iastate.edu, jdekkers@iastate.edu, trkelly@ucdavis.edu, hzhou@ucdavis.edu

## Slide 2
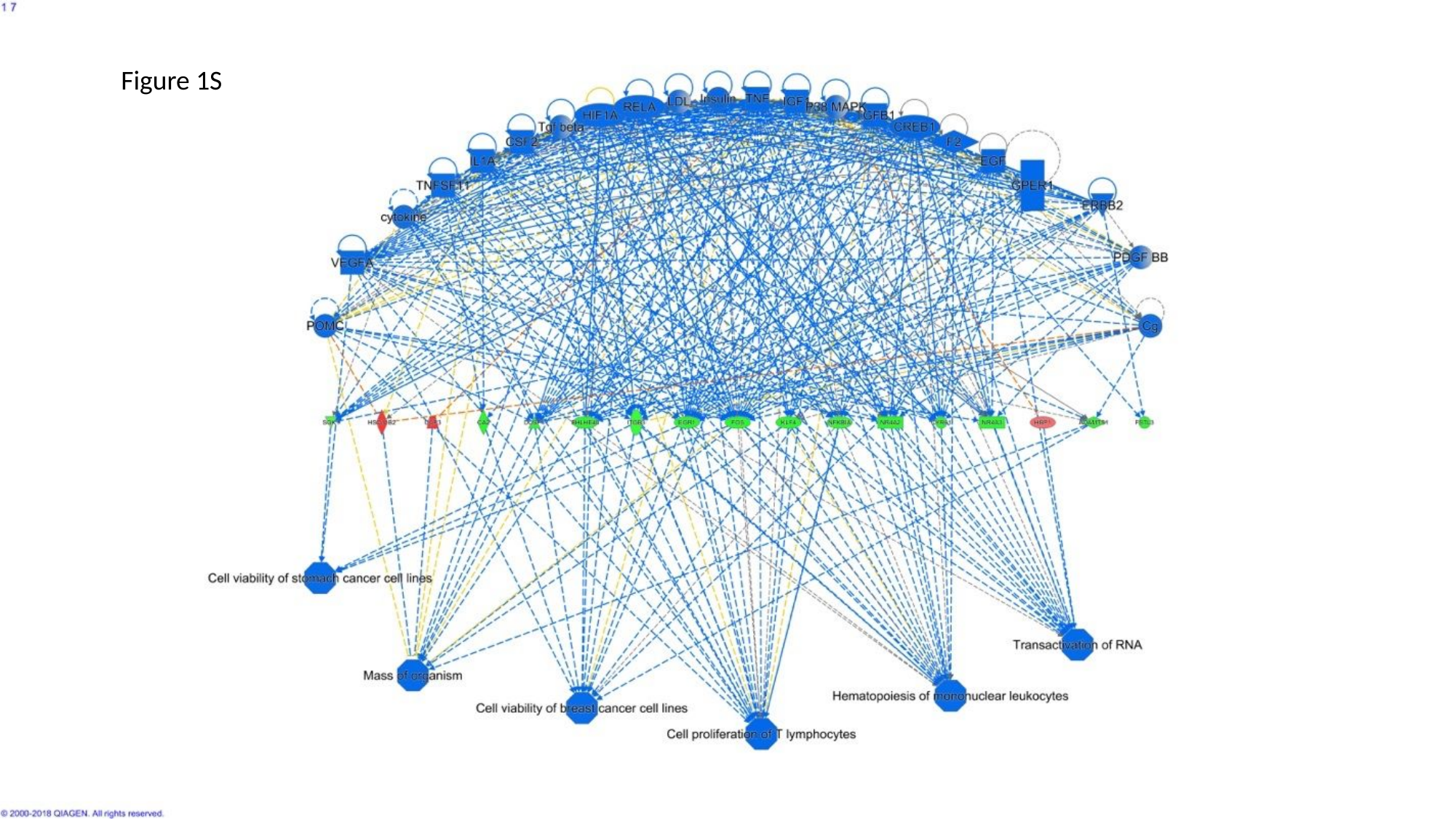

Figure 1S

## Slide 3
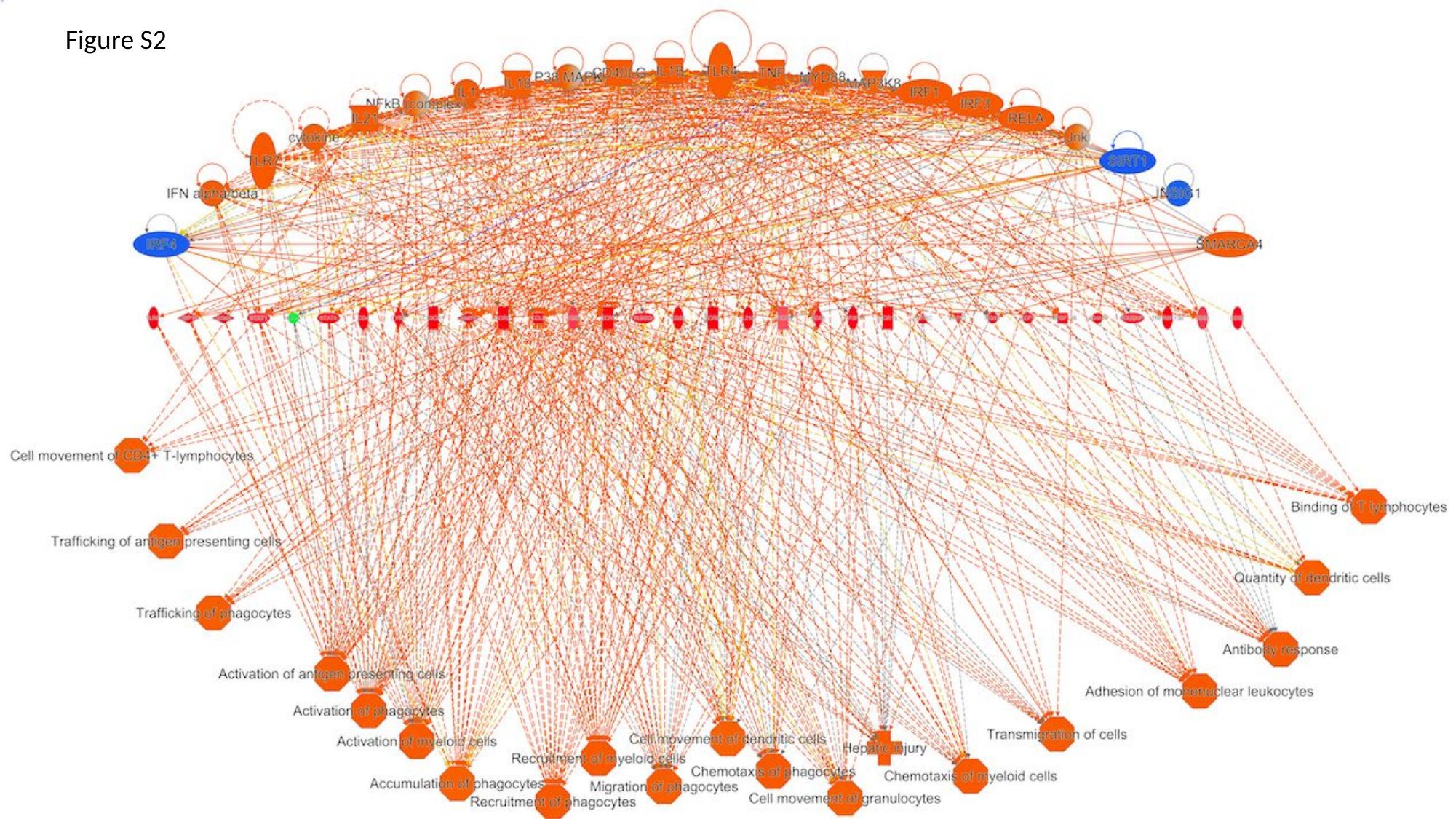

Figure S2
